# Identification of Late Larval Stage Developmental Checkpoints in *Caenorhabditis elegans* Regulated by Insulin/IGF and Steroid Hormone Signaling Pathways

**DOI:** 10.1371/journal.pgen.1004426

**Published:** 2014-06-19

**Authors:** Adam J. Schindler, L. Ryan Baugh, David R. Sherwood

**Affiliations:** Department of Biology, Duke University, Durham, North Carolina, United States of America; University of California San Francisco, United States of America

## Abstract

Organisms in the wild develop with varying food availability. During periods of nutritional scarcity, development may slow or arrest until conditions improve. The ability to modulate developmental programs in response to poor nutritional conditions requires a means of sensing the changing nutritional environment and limiting tissue growth. The mechanisms by which organisms accomplish this adaptation are not well understood. We sought to study this question by examining the effects of nutrient deprivation on *Caenorhabditis elegans* development during the late larval stages, L3 and L4, a period of extensive tissue growth and morphogenesis. By removing animals from food at different times, we show here that specific checkpoints exist in the early L3 and early L4 stages that systemically arrest the development of diverse tissues and cellular processes. These checkpoints occur once in each larval stage after molting and prior to initiation of the subsequent molting cycle. DAF-2, the insulin/insulin-like growth factor receptor, regulates passage through the L3 and L4 checkpoints in response to nutrition. The FOXO transcription factor DAF-16, a major target of insulin-like signaling, functions cell-nonautonomously in the hypodermis (skin) to arrest developmental upon nutrient removal. The effects of DAF-16 on progression through the L3 and L4 stages are mediated by DAF-9, a cytochrome P450 ortholog involved in the production of *C. elegans* steroid hormones. Our results identify a novel mode of *C. elegans* growth in which development progresses from one checkpoint to the next. At each checkpoint, nutritional conditions determine whether animals remain arrested or continue development to the next checkpoint.

## Author Summary

Organisms in the wild often face long periods in which food is scarce. This may occur due to seasonal effects, loss of territory, or changes in predator-to-prey ratio. During periods of scarcity, organisms undergo adaptations to conserve resources and prolong survival. When nutrient deprivation occurs during development, physical growth and maturation to adulthood is delayed. These effects are also observed in malnourished individuals, who are smaller and reach puberty at later ages. Developmental arrest in response to nutrient scarcity requires a means of sensing changing nutrient conditions and coordinating an organism-wide response. How this occurs is not well understood. We assessed the developmental response to nutrient withdrawal in the nematode worm *Caenorhabditis elegans*. By removing food in the late larval stages, a period of extensive tissue formation, we have uncovered previously unknown checkpoints that occur at precise times in development. Diverse tissues and cellular processes arrest at the checkpoints. Insulin-like signaling and steroid hormone signaling regulate tissue arrest following nutrient withdrawal. These pathways are conserved in mammals and are linked to growth processes and diseases. Given that the pathways that respond to nutrition are conserved in animals, it is possible that similar checkpoints may also be important in human development.

## Introduction

The development of multicellular organisms requires the coordinated differentiation and morphogenesis of multiple cell types that interact to form functional tissues and organs. In favorable environmental conditions, development proceeds in a largely stereotyped pattern. When faced with adverse conditions, tissue growth may slow or arrest until the environment improves [1]–[3]. The most critical environmental factor that regulates development is nutrient availability. Organisms can modulate growth programs in response to changing nutritional conditions [4], although the mechanisms through which organisms sense changes in nutrient availability and alter diverse cellular processes in a coordinated manner are incompletely understood.

The nematode *Caenorhabditis elegans* is a powerful model for understanding the effects of nutrition on development due to its short life cycle (3–4 days from embryo to adult), simple cellular make-up, and highly stereotyped development. The postembryonic development of *C. elegans* entails passage through four larval stages (L1–L4) that are separated by molts, before reaching reproductive adulthood. Two alternative pathways of development exist in *C. elegans*: continuous passage through the four larval stages, or entry into an L3 dauer stage, a growth-arrested state characterized by altered body morphology, elevated stress resistance, and prolonged survival [3]. Entry into dauer is initiated late in the L1 stage in response to unfavorable environmental conditions, in particular high population density, high temperature, and reduced nutrient availability [5]. Additional points of arrest in response to poor nutritional conditions have been identified early in the *C. elegans* life cycle and in adults. Animals that hatch in the absence of food undergo L1 arrest [6], [7], and animals reared from hatching on a limited supply of heat-killed bacteria arrest in the L2 stage [8]. Finally, adult *C. elegans* arrest embryo production and shrink their germlines following removal of food [9], [10].

Studies on dauer and L1 arrest have revealed critical roles for the insulin/insulin-like growth factor (IGF) signaling pathway in sensing the nutritional environment and regulating entry into arrest [7], [11], [12]. In *C. elegans*, insulin-like peptides are generated during feeding and signal through DAF-2, the insulin/IGF receptor. Activation of DAF-2 leads to the phosphorylation and cytoplasmic sequestration of DAF-16, a forkhead box O (FOXO) transcription factor. During conditions of low nutrition, the DAF-2-mediated phosphorylation of DAF-16 is reduced, allowing DAF-16 to enter the nucleus and transcriptionally regulate genes implicated in developmental arrest [7], [13]–[16]. Mutant animals with reduced *daf-2* function may arrest in the L1 stage or form dauers constitutively (Daf-c phenotype) [11], whereas *daf-16* null mutants continue development past the wild type timing of L1 arrest and are defective in dauer formation (Daf-d phenotype) [2], [12].

In worms, insects, and mammals, insulin-like signaling affects the production of steroid hormones, lipophilic molecules that bind to nuclear hormone receptors and induce cellular responses [17]. In *C. elegans*, bypassing dauer formation requires the bile-acid like steroid hormone dafachronic acid (DA) [18]. DAF-9, a cytochrome P450 ortholog, is required for the production of DAs, and *daf-9* null animals are Daf-c [19]–[21]. The effects of DAF-9 on dauer formation are mediated by DAF-12, a nuclear hormone receptor that binds DAs [18]. The steroid hormone pathway functions downstream of insulin-like signaling during dauer formation, as *daf-12* Daf-d alleles suppress the Daf-c phenotype of *daf-2* mutants [11].

Despite extensive work on dauer and other arrests, questions remain about the response of *C. elegans* to nutritional scarcity. Among these are whether the arrests in L1, L2, dauer, and the adult represent unique periods of the life cycle during which animals are sensitive to their nutritional environment, or if arrest can also occur at other times. It is also not known whether bypassing the opportunity to form a dauer leads to continuous development to adulthood, or whether additional opportunities exist to arrest development when faced with nutrient deprivation. Finally, the mechanisms through which numerous tissues and cellular processes are able to coordinately arrest in response to nutrient withdrawal are not well understood.

We sought to address these questions by examining the response of *C. elegans* to nutrient deprivation during the late larval stages (L3 and L4), after the opportunity to form a dauer has been passed. Several tissues that contribute to the reproductive system undergo differentiation, growth, and morphogenesis during the L3 and L4 stages, making this period an ideal time to determine how ongoing developmental processes are affected by nutrient deprivation. By removing animals from food at different times, we show that specific checkpoints exist in the early part of the L3 and L4 stages that restrict progression through the larval stage and systemically arrest the development of diverse tissues and cellular processes. Insulin-like signaling regulates the response to nutrient deprivation in the L3 and L4 stages through cell-nonautonomous DAF-16 activity in the hypodermis (skin), and functions to suppress DAF-9–mediated signaling activity. Our results identify a mode of metazoan growth in which development proceeds from checkpoint to checkpoint. At these checkpoints, nutritional conditions determine whether animals remain in an arrested state or continue development to the next checkpoint.

## Results

### Overview of vulval development in *C. elegans*

**Figure 1:**
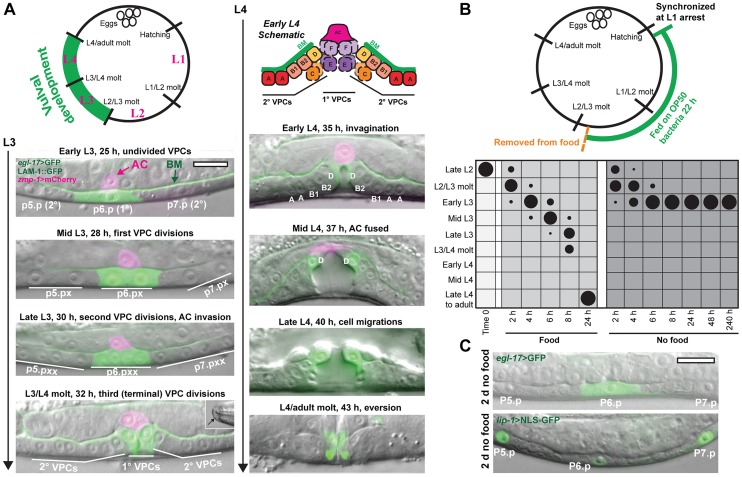
Removal from food induces arrest in vulval development early in the L3 stage. (A) Vulva development in the L3 and L4 larval stages. The 1°-fated vulval precursor cell (VPC), P6.p, expresses *egl-17*>GFP in green; P5.p and P7.p are specified to the 2° VPC fate. Basement membrane (BM; expressing LAM-1::GFP protein in green) separates the uterine and vulval epithelium. The uterine anchor cell (AC; expressing *zmp-1*>mCherry in magenta) is dorsal to P6.p and invades across BM between the first and second VPC divisions. The final VPC divisions occur at the time of the L3/L4 molt. Molting animals can be distinguished by the formation of buccal caps covering the mouth (inset, bottom left panel). (Top right of panel A) Cell divisions produce 22 VPC progeny that comprise seven vulval subtypes, vulA–vulF. Some of the cells divide along the left-right axis (hatched lines in early L4 schematic) outside the central plane of focus. In the mid L4 stage, the AC fuses with the surrounding uterine cells, and *egl-17*>GFP expression changes from the 1° to 2°-fated cells. At the end of L4, the cells turn partially inside out (evert). Times for each developmental stage are after release from L1 arrest at 20°C. (B) Late L2 nutrient deprivation assay. Animals were removed from food after 22 h growth at 20°C and either returned to food or kept deprived of food. Starting at time 0, both groups were maintained at RT (22°C). In the chart, developmental stages on the Y-axis were determined by the extent of vulval development and the molt, and the duration of feeding or removal from food is indicated on the X-axis. The areas of the circles in the chart reflect the percentage of the population at each stage of development; n≥50 for each time point. See Fig. S1 for raw data and results of replicate assays. (C) Animals after 2 d removal from food, with no divisions of P5.p–P7.p, and expressing the 1° fate marker, *egl-17*>GFP (top), or the 2° fate marker *lip-1*>NLS-GFP (bottom). In these and other figures, anterior is left. Scale bars, 10 µm.

To study the effects of nutrient deprivation on tissue development during the late larval stages, we first focused on the hermaphrodite vulva, which develops through a stereotyped pattern of cell specification, cell division, and morphogenesis during the L3 and L4 stages (Fig. 1A) [22]. The vulva derives from three epidermal cells, P5.p–P7.p, which are specified early in the L3 stage to either the 1° vulval precursor cell (VPC) fate (P6.p) or the 2° VPC fate (P5.p and P7.p) (Fig. 1A). The VPCs undergo three rounds of cell division in the L3 stage to generate 22 cells, which differentiate into seven vulval subtypes, vulA–vulF. In the L4 stage, the 22 vulval cells undergo morphogenetic processes that include invagination, migration, and cell-cell fusion (Fig. 1A) [22]. Proper development of the vulva requires the uterine anchor cell (AC), which invades in the mid L3 stage across basement membranes separating the uterine and vulval epithelia to form a connection between the tissues [23]. The AC remains at the vulval apex after invasion until fusing with surrounding uterine cells in the mid L4 stage (Fig. 1A).

### Vulval development arrests early in the L3 larval stage after removal of food

We examined the effects of nutrient deprivation on vulval development by growing a synchronized population to late in the L2 stage, prior to the onset of vulval formation, and removing animals from food (Fig. 1B). Part of the population was returned to food to serve as controls, with the remainder maintained in M9, a buffer lacking a carbon source. In addition to the vulva, we also assessed the onset of molting (observable by cuticle covering the mouth; see Fig. 1A, bottom left), which serves as a marker for the transition between larval stages. Results of the experiment are depicted graphically in Fig. 1B; raw data and results of triplicate assays are in Fig. S1. The control group that was returned to food progressed through the stages of vulval development with the predicted timing. The group that remained deprived of food molted into the L3 stage and uniformly arrested prior to the first VPC divisions. No VPC divisions were observed after 10 days in the absence of food (Fig. 1B). Arrested animals were phenotypically distinct from dauer larvae, which arrest after molting into a specialized L3 dauer stage (Fig. S2). When animals were returned to food after 8 d, 97.5% of the population (n = 200) continued development to adulthood, demonstrating that animals retain the capacity to resume development upon re-introduction of food. The median survival of animals under the experimental conditions used was 11.7±1.2 d (n = 3 trials).

In *C. elegans*, removal of the germline either through ablation or genetic mutation extends lifespan and maintains adult somatic tissues for a longer duration in a youthful state [24]–[26]. This suggests the possibility of a soma-germline tradeoff in which resources are allocated away from germ cells to somatic tissues. We asked if the absence of a germline could promote the continued development of somatic tissues by growing *glp-1(e2144)* mutants, which do not proliferate germline progenitor cells when reared at 25°C, to late in the L2 stage and removing them from food. We found no difference in the timing of arrest, as all *glp-1(e2144)* animals (n = 100) arrested prior to VPC divisions. These results demonstrate that the absence of the germline does not alter the timing of somatic tissue arrest in response to nutrient removal.

In addition to cell divisions, fate specification of the vulval cells was also examined in L3-arrested animals. Vulval fates are specified between the late L2 and early L3 stages, when an inductive LIN-3/EGF signal from the AC and lateral LIN-12/NOTCH signaling between VPCs combine to specify the 1° fate in P6.p and 2° fates in P5.p and P7.p [27], [28]. To determine the state of VPC specification in arrested animals, we examined a marker of 1° fate, *egl-17*>GFP [29], and a marker of 2° fate, *lip-1*>NLS-GFP [30] (see Materials and Methods for description of transgene nomenclature). All arrested animals expressed *egl-17*>GFP exclusively in P6.p, and 93% of animals expressed *lip-1*>NLS-GFP at elevated levels in P5.p and P7.p (n = 30 per assay; Fig. 1C), demonstrating that arrest occurred after 1° and 2° VPC specification. This contrasts with dauer larvae, which are not stably specified to a VPC fate [31], [32]. Coupled with the absence of VPC divisions, these results suggest that, when removed from food late in the L2 stage, vulval development arrests early in the L3 stage in a manner that is distinct from dauer arrest.

### A second arrest point in vulval development occurs early in the L4 stage

**Figure 2:**
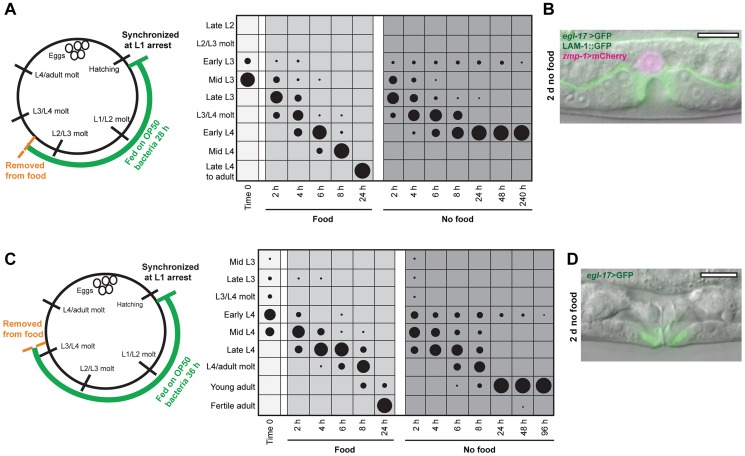
Vulval development arrests at a precise time in the L4 stage. (A) Schematic and chart for mid L3 nutrient removal assay. A wild type population was grown for 28 h at 20°C and removed from food. Stages of vulval development were assessed in fed and nutrient deprived groups as described in Fig. 1B; n≥50 for each time point. (B) Image of L4-arrested animal 48 h after removal from food. *egl-17*>GFP was expressed exclusively in 1° VPC progeny and VPC divisions had completed. The AC, expressing *zmp-1*>mCherry, invaded across basement membrane but did not fuse with the surrounding uterine cells. (C) Schematic of early L4 nutrient deprivation assay, with animals grown for 36 h at 20°C and scored for developmental stage as described for Figs. 1B and 2A. See Fig. S1 for raw data and replicates of assays in (A) and (C). (D) Adult animal after 2 d removal from food, with eversion of the vulva. Scale bars, 10 µm.

The uniform arrest of vulval development early in the L3 stage suggested that a specific developmental checkpoint existed at this time. To determine if this was the case, we asked whether vulval development could arrest at later times in the L3 stage. A synchronized population was grown for 28 h to the mid L3 stage and removed from food. At the time of food removal, 84% had undergone one VPC division, indicating that they had bypassed the L3 arrest point (Fig. 2A; Fig. S1). Animals removed from food continued through the L3 stage, molted into L4, and arrested in L4 after completion of VPC divisions (Fig. 2A). After 48 h in the absence of food, 94% of the population was arrested after VPC divisions in the L4 stage. The remaining 6% of animals was arrested in the L3 stage prior to VPC divisions, and likely represent the youngest members of the population that failed to bypass early L3 arrest. No animals were identified at intermediate stages between the two arrest points, demonstrating that bypass of the L3 arrest point led to invariant progression to the L4 arrest point (Fig. 2A). We examined the effect of the germline on L4 arrest by removing *glp-1(e2144)* mutants grown at 25°C from food in the mid L3 stage, and found that all animals arrested in early L4 similar to wild type (n = 100). The median survival of populations removed in the mid L3 stage was 11.0 d (n = 3 trials).

All L4-arrested animals completed vulval cell divisions (n = 30), suggesting that arrest in vulval formation could occur at a precise developmental time rather than in a variable manner. To test this notion, we first examined the reporter gene *egl-17*>GFP, which is expressed in 1° VPC progeny early in the L4 stage and shifts to 2° VPC progeny in mid L4 (Fig. 1A). Expression of *egl-17*>GFP was exclusively in 1° VPC progeny in arrested animals (Fig. 2B), supporting the hypothesis of a precise timing of arrest early in the L4 stage. A second marker for L4 stage timing in vulval development is cell-cell fusions. Fusions occur between homotypic cells (i.e., vulA with vulA), starting with vulA cells shortly after terminal cell divisions and continuing two hours later with vulC cells (Fig. S3) [33]. Examination of a strain expressing GFP-tagged AJM-1, an apical-membrane–localized protein that delineates the boundaries of vulval cells [33], [34], showed that all L4-arrested animals had undergone vulA fusions but not vulC fusions (Fig. S3). Importantly, the same timing of arrest between vulA and vulC fusions occurred in 97% of the population when animals were grown for an additional four hours prior to removal from food (n = 30 per assay; Fig. S3), demonstrating that the timing of arrest in vulval development in the L4 stage is largely independent of feeding duration. Based on the nutrient removal experiments, we conclude that specific checkpoints exist early in the L3 and L4 larval stages that arrest vulval development at precise developmental times.

Only a single checkpoint on vulval development was identified in the L3 stage, and we wanted to determine whether this was also the case with the L4 stage. Animals were grown on food to the early-to-mid L4 stage and developmental progression examined following food removal (Fig. 2C; Fig. S1). After 48 h in the absence of food, 96% of the population had progressed into adulthood, as evidenced by eversion of the vulva (Fig. 2D), with the remaining animals arrested early in the L4 stage, and no animals at intermediate times (Fig. 2C). Arrest occurred in nearly all adult animals (99/100) prior to oogenesis. Taken together, the nutrient removal assays show that arrest in *C. elegans* vulval development occurs only at precise checkpoints early in the L3 and L4 stages, and that passage through one checkpoint leads invariantly to progression through the larval stage to the next checkpoint.

### A cell-nonautonomous developmental mechanism regulates the timing of arrest

**Figure 3:**
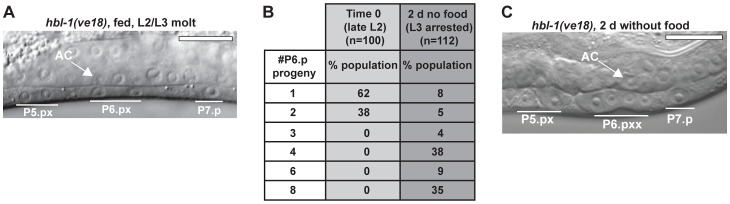
A systemic mechanism coordinates the timing of L3 arrest. (A) Precocious VPC divisions in *hbl-1(ve18)* mutant animals. At the time of the L2/L3 molt, P5.p and P6.p have undergone cell divisions. (B) Variable number of P6.p progeny after 2 d removal from food. The number of P6.p progeny was counted when food was removed late in the L2 stage, and again after 2 d. (C) Image of *hbl-1(ve18)* after 2 d removal from food, with four P6.p progeny and two P5.p progeny. The AC (signified by white arrow) has not breached the basement membrane, indicative of arrest early in the L3 stage. Scale bars, 10 µm.

There are two alternative possibilities for the timing of arrests observed in vulval formation in the L3 and L4 stages. The first is of a tissue-autonomous program in which arrests occur only at specific times in the developmental program, either prior to cell divisions (early L3) or after cell divisions (early L4). The second is of a global timing mechanism that arrests vulval development at precise times early in each larval stage. To determine which of these was correct, we examined *hbl-1(ve18)* mutant animals, which have precocious VPC divisions that occur as early as the late L2 stage (Fig. 3A) [35]. We hypothesized that if the vulval cells were regulated by an autonomous program, then shifting the time of cell divisions relative to the L3 larval stage would not affect the all-or-none pattern of cell divisions. If instead a global timing mechanism directed the arrest of vulval development, then cell divisions would be predicted to arrest upon reaching the L3 larval stage checkpoint. Results of the experiment show that after removal from food late in the L2 stage, P6.p divisions continued but stopped prior to completion (Fig. 3B–C). Only 43% of the population was arrested either prior to or after cell divisions, with the remainder at intermediate stages of division (Fig. 3B). These results support the idea of a global timing mechanism that acts on vulval development to arrest it at specific times early in the larval stage.

### Arrest occurs at a precise time in the larval stage and molting cycle

**Figure 4:**
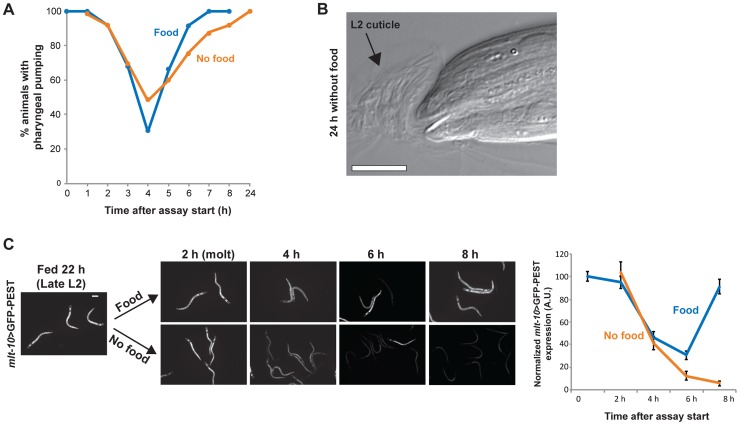
Arrest occurs at a specific time in the larval stage and molting cycle. (A) Percentage of animals with pharyngeal pumping in fed and nutrient-deprived groups maintained at 22°C; n≥50 at each time point per group. Absence of pharyngeal pumping indicates that animals are in lethargus. (B) An L3-arrested animal having completed ecdysis of the L2 cuticle, after 24 h in the absence of food. The shed cuticle surrounds the head. (C) Images of fed and nutrient-deprived animals expressing the molting cycle reporter gene *mlt-10*>GFP-PEST. Chart shows quantification of *mlt-10*>GFP-PEST expression levels over an 8 h interval starting late in the L2 stage. Error bars ± S.D. for n≥30 at each time point. Scale bars, 10 µm.

The experiments on vulval development suggested that checkpoints exist at particular points in the larval stage. We wanted to explore this question in more detail by examining progression through the larval stage in the absence of food. Each *C. elegans* larval stage comprises a period of foraging for food that lasts for several hours, followed by an approximately two-hour period of lethargus during which pharyngeal pumping stops and animals do not feed. At the end of lethargus, *C. elegans* undergo molting, the detachment (apolysis) and shedding (ecdysis) of the existing cuticle [36]. We first asked if animals removed from food during the period of foraging underwent lethargus, and found that both the onset and duration of lethargus were similar to a control population that was maintained on food. Further, animals exited lethargus and resumed pharyngeal pumping for at least 24 h after removal from food (Fig. 4A). These results show that lethargus, a key feature of the larval stage, is maintained in the absence of food.

We next examined how nutrient deprivation affected the molting cycle, the oscillatory pattern of gene expression and cuticle replacement that occurs in each larval stage. Cuticle components are synthesized starting in the mid-larval stage and deposited underneath the existing cuticle, which is shed at the end of the larval stage [37], [38]. The timing of the checkpoints in the early part of larval stage suggested that arrest could occur after molting and prior to new cuticle synthesis. We first asked whether this was the case by examining the execution of the molt following removal of food. We found that all L3- and L4-arrested animals completed ecdysis (Fig. 4B), although 17% of adult-arrested animals remained attached to the L4 cuticle after 48 h in the absence of food (n = 100 animals per assay). It is possible that larger animals may not be able to fully shed cuticle in the absence of sufficient feeding. Despite this defect, animals were viable and resumed pharyngeal pumping, with the L4 cuticle remaining attached only in the tail region. These results demonstrate that molting is successfully executed in most instances upon passage through a checkpoint.

We then explored the second part of our hypothesis that arrest occurred prior to new cuticle synthesis. To do this, we examined the expression pattern of *mlt-10*, a gene required for proper execution of the molt [38], [39]. *mlt-10* mRNA increases in the mid-larval stage at the time of new cuticle synthesis, peaks during the molt, and declines upon completion of molting. A destabilized reporter gene, *mlt-10*>GFP-PEST, recapitulates this oscillatory mRNA expression pattern and serves as a marker for progression through the larval stage [38], [39]. A population of *mlt-10*>GFP-PEST–expressing animals was removed from food late in the L2 stage and a portion of the population returned to food to serve as controls. The fed and nutrient-deprived groups were then examined for reporter gene expression over an 8 h period. Results show that expression levels were similar in the two groups as they molted and entered the L3 stage (Fig. 4C). Approximately 4 h after molting, the control group increased gene expression, indicating initiation of the L3 molting cycle. The nutrient-deprived group failed to increase expression, however, demonstrating that it had arrested prior to initiation of the L3 molting cycle (Fig. 4C). Similar results were observed when animals were removed from food late in the L3 stage (data not shown). The loss of *mlt-10*>GFP-PEST expression was unlikely to be due to general transcriptional silencing during nutrient deprivation, as past research has shown that several transcriptional reporters similarly tagged with PEST motifs maintain expression during L1 arrest [40]. Collectively, these results demonstrate that *C. elegans* arrest development during the L3 and L4 stages at a specific point after molting and prior to new cuticle synthesis.

### Feeding is required after molting to bypass the L3 and L4 checkpoints

Our results identified nutrient-sensitive developmental checkpoints in the early part of the L3 and L4 larval stages. We sought to determine the amount of feeding required to pass the checkpoints. To achieve the greatest degree of synchronization and most accurate measurement of timing, individual animals were isolated during ecdysis, the final 10–15 minutes of molting that precede foraging [36]. Animals undergoing ecdysis were either removed from food or allowed to feed for additional 30 min intervals (Fig. S4). Feeding for 30–60 min after ecdysis was required for most animals to pass the L3 and L4 checkpoints within 24 h after food removal, and 90 minutes of feeding resulted in all animals passing the checkpoints (Fig. S4). We conclude that a sufficient duration of feeding is required after molting to advance past the L3 and L4 stage checkpoints.

### Insulin-like signaling regulates the response to nutritional conditions in the L3 and L4 stages

**Figure 5:**
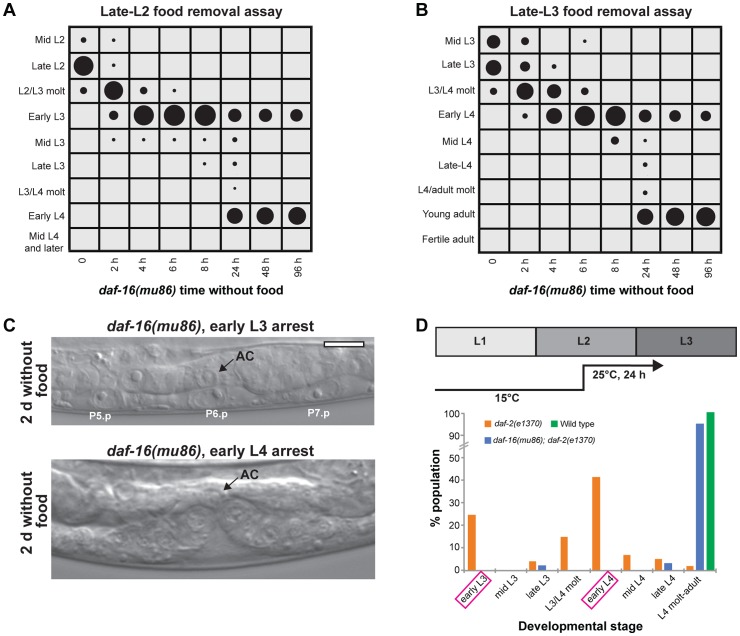
The insulin-like signaling pathway regulates progression past the L3 and L4 checkpoints. (A) *daf-16(mu86)* animals were grown to late in the L2 stage, removed from food, and the stage of development assessed at intervals using vulval development and the molt as markers; n≥50 at each time point. (B) Similar assay as in (A), with *daf-16(mu86)* animals grown to late in the L3 stage. See Fig. S5 for raw data and replicates of assays in (A) and (B). (C) Images of *daf-16(mu86)* animals after 2 d removal from food and arrested in the early L3 (top) or early L4 (bottom) stages. No VPC divisions were observed in L3-arrested animals, and VPCs completed divisions in L4-arrested animals. (D) Wild type, *daf-2(e1370), and daf-2(e1370); daf-16(mu86)* animals were fed to the mid L2 stage at 15°C and shifted to 25°C. After 24 h additional feeding at 25°C, the developmental stage was examined for n = 100 animals per genotype. In the presence of food, *daf-2(e1370)* animals paused preferentially early in the L3 and L4 stages (highlighted in magenta). Scale bar, 10 µm.

The insulin-like signaling pathway is a key regulator of growth in response to nutrition [41]. We wanted to determine if insulin-like signaling regulated arrest in the L3 and L4 stages following nutrient removal. We first asked if *daf-16*, a FOXO transcription factor that is a major target of insulin-like signaling and is required for the proper timing of L1 arrest and dauer formation [7], [12], played a role in L3 and L4 arrest. Animals with the null mutation *daf-16(mu86)* were removed from food late in the L2 stage, and the developmental stage assessed over time by examination of the vulva and molt. The pattern of growth by 8 h after food removal was similar to wild type: all animals had molted into L3 and 97% were in the early L3 stage (Fig. 5A; raw data and results of replicate assays are in Fig. S5). By 24 h after removal from food, however, 63% of the population had progressed past the L3 checkpoint (compared with 0% of wild type animals removed from food at a similar time; Fig. 1B), ultimately arresting early in the L4 stage (Fig. 5A). A second experiment was performed with *daf-16(mu86)* animals removed from food late in the L3 stage. Again, the absence of *daf-16* caused animals to bypass arrest, with 72% of the population progressing to adulthood after 48 h (Fig. 5B; Fig. S5). The time in the larval stage at which *daf-16(mu86)* animals arrested was similar to wild type, based on the absence of VPC divisions in L3-arrested animals, the completion of divisions in L4-arrested animals, and no animals at intermediate stages of division (n = 30; Fig. 5C).

In the presence of food, DAF-16 activity is inhibited by a signaling pathway downstream of DAF-2, the insulin/IGF receptor. We hypothesized that animals with reduced DAF-2 function would require a longer duration of feeding to inhibit DAF-16 activity and progress through the L3 and L4 larval stages. To test this hypothesis, we examined the L3 and L4 development of a temperature-sensitive *daf-2* mutant, *daf-2(e1370)*, which is Daf-c at 25°C but develops to adulthood at 15°C [11]. Animals were grown at the permissive temperature of 15°C to the mid-L2 stage, bypassing the opportunity to form a dauer, and shifted to the restrictive temperature of 25°C for an additional 24 h feeding (Fig. 5D). Following this regimen, 25% of the population was in the early L3 stage and 42% was in early L4 stage. In contrast, a control wild type population had progressed to the L4/adult molt or beyond (Fig. 5D). The high proportion of the population in the early L3 and early L4 stages suggests that prolonged pausing at the checkpoints may be a factor in the delayed development of *daf-2(e1370)* animals. This delayed development required the presence of *daf-16*, as *daf-16(mu86); daf-2(e170)* double mutant animals advanced through the L3 and L4 stages at a rate comparable to wild type (Fig. 5D). Taken together, the results of the *daf-16* and *daf-2* experiments demonstrate a role for the insulin-like signaling pathway in regulating progression through the L3 and L4 developmental arrest checkpoints in response to nutritional conditions.

### DAF-16 functions cell-nonautonomously in the hypodermis to mediate L3 and L4 arrest

**Figure 6:**
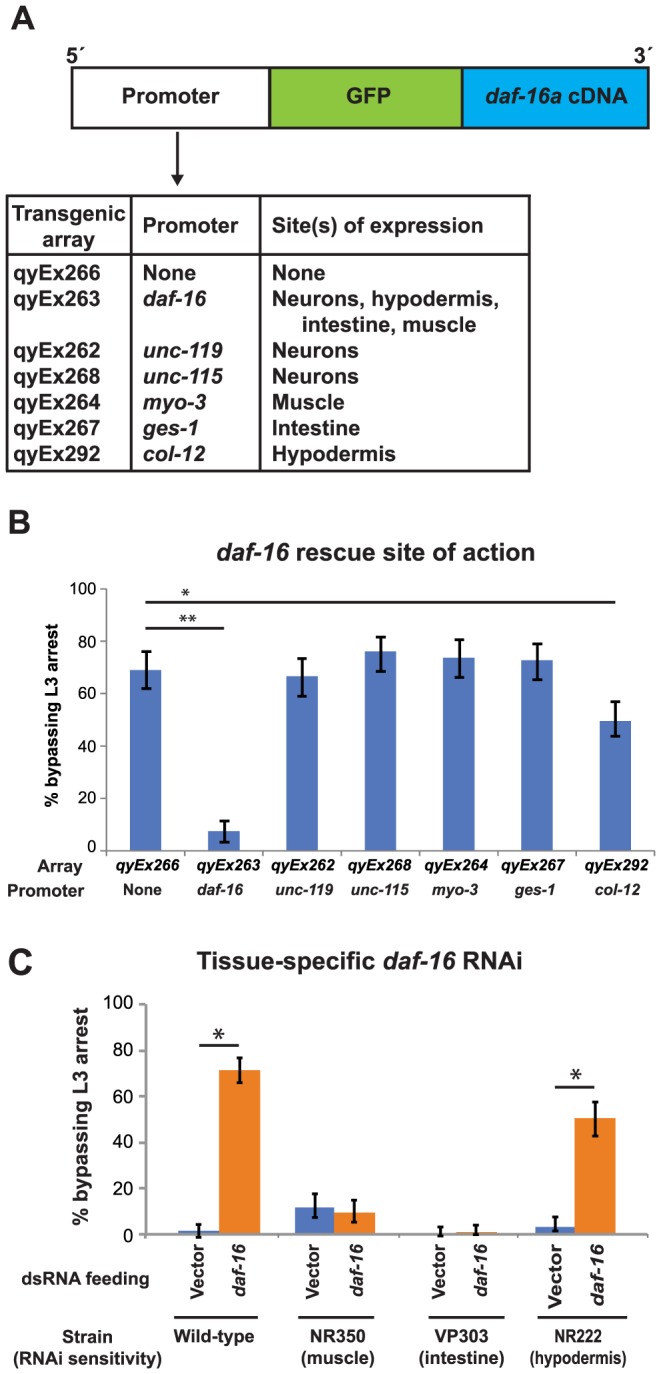
Expression of DAF-16 in the hypodermis regulates the L3 nutritional response. (A) A schematic diagram of transgenes tested for rescue of the *daf-16(mu86)* bypass phenotype and their sites of expression (see also Supplemental Fig. 6). (B) *daf-16(mu86)* animals expressing the transgenic arrays in (A) were assayed for bypass of L3 arrest. Averages of 3 assays; n≥50 per assay. Error bars denote 95% confidence interval; *p<.0001, **p<.001 by two-tailed Fisher's exact test. (C) Wild type and tissue-specific RNAi sensitive strains (see Experimental Procedures for descriptions) were fed either L4440 (empty vector control) or *daf-16* dsRNA, and the percentage of animals bypassing L3 arrest were measured 2 d after removal from food. Average of 5 assays (wild type) or 3 assays (all others); n≥50 per assay. Error bars denote 95% confidence interval; *p<.0001 by two-tailed Fisher's exact test.

Previous work has shown that DAF-16 functions cell-nonautonomously to regulate multiple physiological processes, including dauer formation, lifespan extension, germline proliferation, and metabolism [42]–[44]. We asked if DAF-16 similarly functioned cell-nonautonomously to regulate L3 and L4 arrest. Plasmids that contained *daf-16* cDNA tagged at the N-terminus with GFP and expressed under the *daf-16* or tissue-specific promoters [42] (Fig. 6A) were injected into *daf-16(mu86); unc-119(ed4)* double mutant animals along with an *unc-119* rescue plasmid. Animals harboring an extrachromosomal array of the plasmids were identified by rescue of the *unc-119(ed4)* movement defect and validated by examination of GFP expression (Fig. S6).

When expressed from the *daf-16* promoter, GFP::DAF-16 protein localized to neurons, hypodermis, intestine, and body wall muscles (Fig. S6), and rescued the *daf-16(mu86)* phenotype, in which animals bypass L3 arrest and continue development to the L4 stage (Fig. 6B). GFP::DAF-16 expressed from tissue-specific promoters for neurons (*unc-119* and *unc-115*), muscle (*myo-3*), and intestine (*ges-1*) [42] failed to rescue the *daf-16(mu86)* bypass phenotype. Only GFP::DAF-16 expression from a hypodermis-specific promoter (*col-12*) significantly rescued the *daf-16(mu86)* phenotype (Fig. 6B). Similar results were obtained in assays examining bypass of L4 arrest (data not shown). The lower efficiency of rescue by *col-12*>GFP::DAF-16 compared to *daf-16*>GFP::DAF-16 could be due to reduced expression of the transgene following removal from food: *col-12*>GFP::DAF-16 expression decreased 71% following 2 d in the absence of food, whereas *daf-16*>GFP::DAF-16 expression increased more than twofold during this time (Fig. S7). As in other assays, vulval development was used as the primary marker for developmental stage. Animals that were rescued for the L3 bypass phenotype by *daf-16*>GFP::DAF-16 or *col-12*>GFP::DAF-16 did not have detectable GFP::DAF-16 expression in the vulva, consistent with cell-nonautonomous DAF-16 activity regulating L3 and L4 development.

To complement these studies, we carried out tissue-specific RNAi of *daf-16*. Reducing *daf-16* specifically in the hypodermis reproduced the phenotype of systemic loss of *daf-16*. Targeted reduction of *daf-16* in the intestine or muscle did not alter sensitivity to the removal from food (Fig. 6C). We were unable to reduce *daf-16* specifically in neurons because of the lower sensitivity of this tissue to RNAi [45], and the inability to directly target neurons by RNAi without off-target effects in the hypodermis [46]. Taken together, the results of *daf-16* tissue-specific rescue and RNAi experiments suggest that the hypodermis is a key site of action for the insulin-like signaling pathway in responding to nutritional conditions during the L3 and L4 stages. Our results do not rule out the possibility that DAF-16 functions in other tissues to also regulate L3 and L4 development, either through modulation of hypodermal DAF-16 function or through independent pathways that synergize with hypodermal DAF-16. Previous studies have shown that DAF-16 can function in multiple tissues to regulate dauer formation and metabolism [42], [43], and such a situation could also occur in regulating passage through the L3 and L4 larval stages.

### DAF-9 regulates L3 and L4 developmental progressions downstream of DAF-16

**Figure 7:**
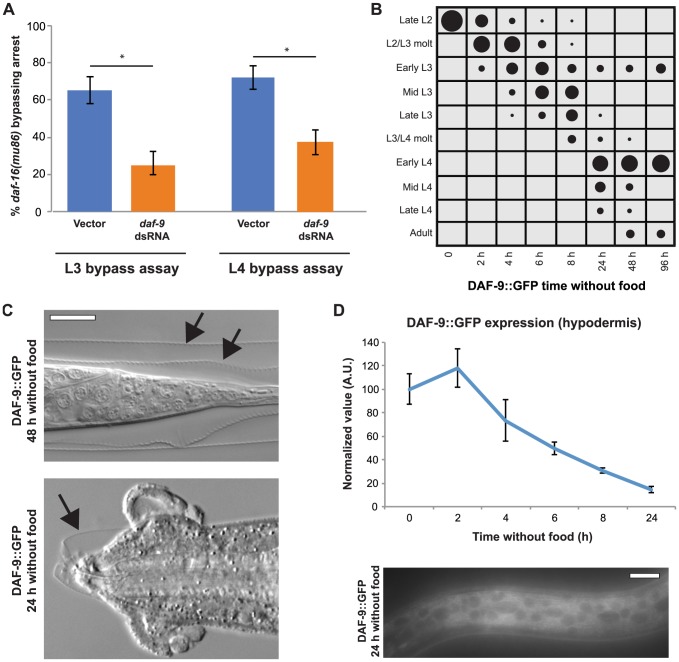
DAF-9 regulates L3 and L4 arrest downstream of DAF-16. (A) *daf-16(mu86)* animals were fed either L4440 (empty vector) or *daf-9* dsRNA and the percentage of animals bypassing L3 and L4 arrest measured 2 d after removal from food. Average of 3 assays; n≥50 per assay. Error bars denote 95% confidence interval; *p<.0001 by two-tailed Fisher's exact test. (B) DAF-9::GFP (*dhIs64*) animals were removed from food late in the L2 stage and developmental progression assessed for n≥50 at each time point. See Fig. S5 for raw data and replicates. (C) Images of adult DAF-9::GFP–expressing animals. Top shows a tail region with both the L3 and L4 cuticles (arrows) still attached. Bottom is a dead or dying animal that has not shed the L4 cuticle surrounding the head (arrow). (D) Normalized expression levels of hypodermal DAF-9::GFP following nutrient deprivation late in the L2 stage. After 24 h, some animals still had detectable levels of the transgene. Error bars ± S.E.M.; n = 20 for each time point. Scale bars, 10 µm.

The ability of DAF-16 to affect tissue development cell-nonautonomously implicated the presence of pathways that signal systemically. One such candidate is DAF-9–mediated steroid hormone signaling, which is downstream of insulin-like signaling during dauer formation [11], [19], [21]. A key site of action for DAF-9 during larval development is the hypodermis [19], [21], [47], suggesting that it could similarly function downstream of insulin-like signaling during the L3 and L4 stages. To test this possibility, we depleted *daf-9* by dsRNA feeding in *daf-16(mu86)* animals, and assessed the response to nutrient removal in the L3 and L4 stages. We hypothesized that, if nuclear DAF-16 inhibited L3 and L4 stage progressions through inhibition of DAF-9–mediated steroid hormone signaling, then the bypass of arrest observed in *daf-16* null animals would be suppressed by reduction of *daf-9*. Consistent with this hypothesis, *daf-9* dsRNA-fed *daf-16(mu86)* animals had a 2.6-fold reduction of bypassing L3 arrest and a 1.9-fold reduction of bypassing L4 arrest compared to empty vector controls (Fig. 7A). These results support the idea that the insulin-like signaling pathway regulates DAF-9–mediated steroid hormone production during the L3 and L4 stages.

Since DAF-9 appeared to be involved in generating hormonal signals that promoted progression through the L3 and L4 larval stages, we asked whether increasing the levels of DAF-9 would lead to bypass of arrest in a manner akin to *daf-16* null animals. This was tested by examining the response to nutrient removal of a strain overexpressing functional *daf-9::GFP* (*dhIs64*) [19]. When removed from food late in the L2 stage, *daf-9*–overexpressing animals bypassed arrest at high levels, with 90% of the population progressed beyond the early L3 stage after 24 h in the absence of food (Fig. 7B; Fig. S5). In contrast to the phenotype of *daf-16(mu86)*, which paused at the L3 checkpoint before bypassing it (Fig. 5A), *daf-9*–overexpressing animals continued past the checkpoint with minimal pausing (Fig. 7B). Further, whereas *daf-16(mu86)* bypassed only one arrest point (Fig. 5A–B), a portion of the *daf-9*-overexpressing population advanced through both the L3 and L4 arrest points and reached adulthood (Fig. 7B). Thus, the bypass of arrest caused by overexpression of *daf-9* is more rapid and robust than that caused by loss of *daf-16*.

*daf-9*–overexpressing animals that progressed to adulthood were typically surrounded by undetached cuticle (41/50 animals); in some cases both the L3 and L4 cuticles remained attached (Fig. 7C). The inability to shed cuticle surrounding the mouth led to lethality in a portion of the population within 24 h of food removal (Fig. 7C). In contrast, neither wild type nor *daf-16* null animals showed such rapid death. These findings demonstrate that overexpression of *daf-9*, which forces animals through larval stages in the absence of food, has deleterious effects on the execution of the molt.

Our finding that *daf-9* overexpression causes continued development in the absence of food were somewhat surprising since a previous study showed that hypodermal DAF-9::GFP expression is sharply reduced during nutrient deprivation [19]. We examined the expression of hypodermal DAF-9::GFP following removal from food late in the L2 stage, and indeed found a reduction in expression over time (Fig. 7D). Expression persisted at low levels in most animals for at least 8 h after removal from food, however, and was visible in some animals even after 24 h (Fig. 7D). These results suggest that, when expressed at elevated levels, enough DAF-9 protein remains in the hypodermis to promote continued larval stage progressions in the absence of food. It is also possible that the second site of DAF-9 expression during larval development, the two neuronal XXX cells, also contribute to the bypass of arrest, as expression is not reduced in those cells following food removal [19]. Collectively, our results offer evidence that DAF-9 promotes passage through the L3 and L4 developmental arrest checkpoints.

### DAF-12 does not regulate L3 and L4 stage progressions

DAF-9 is required for the synthesis of dafachronic acids (DAs), steroid hormones that bind to the nuclear hormone receptor DAF-12 to promote bypass of dauer formation [18]. In the absence of DAs, DAF-12 regulates entry into dauer though its DNA-binding activity [48]. Because our results showed that genes that regulate dauer formation—*daf-2*, *daf-16*, and *daf-9*—also regulate later larval development, we asked if *daf-12* similarly had a role in regulating progression through the L3 and L4 stages downstream of *daf-9*. We first examined the response to nutrient removal of *daf-12(rh61rh411)*, a null mutant that has a Daf-d phenotype [48]. In contrast to *daf-16(mu86)* Daf-d mutants, which bypass L3 arrest over 60% of the time (Fig. 5B), no *daf-12* null mutants bypassed L3 arrest after 48 h in the absence of food (n = 54). We also performed epistasis experiments by generating *daf-12(rh61rh411); daf-16(mu86)* double mutant animals. The bypass phenotype of *daf-16(mu86)* was not suppressed by the loss of *daf-12* function, as 71% of double mutant animals bypassed the L3 checkpoint after 48 h in the absence of food (n = 68), similar to the percentage of *daf-16(mu86)* animals that bypass arrest (Fig. 5B). These results suggest that *daf-16* functions independently of *daf-12* in regulating L3 stage progression. We also asked whether the phenotype of *daf-9* overexpression was suppressed by a null allele of *daf-12*. When removed from food late in the L2 stage, DAF-9::GFP; *daf-12(rh61rh411)* animals still bypassed arrest to a high degree: 87% of the population had bypassed L3 arrest by 24 h (n = 61), similar to the 90% observed in DAF-9::GFP animals (Fig. 7B). These results provide evidence that DAF-12 does not regulate L3 and L4 stage progressions, and that DAF-9 promotes bypass of the L3 and L4 checkpoints through a different downstream effector.

### The L3 and L4 checkpoints arrest tissue development systemically

**Figure 8:**
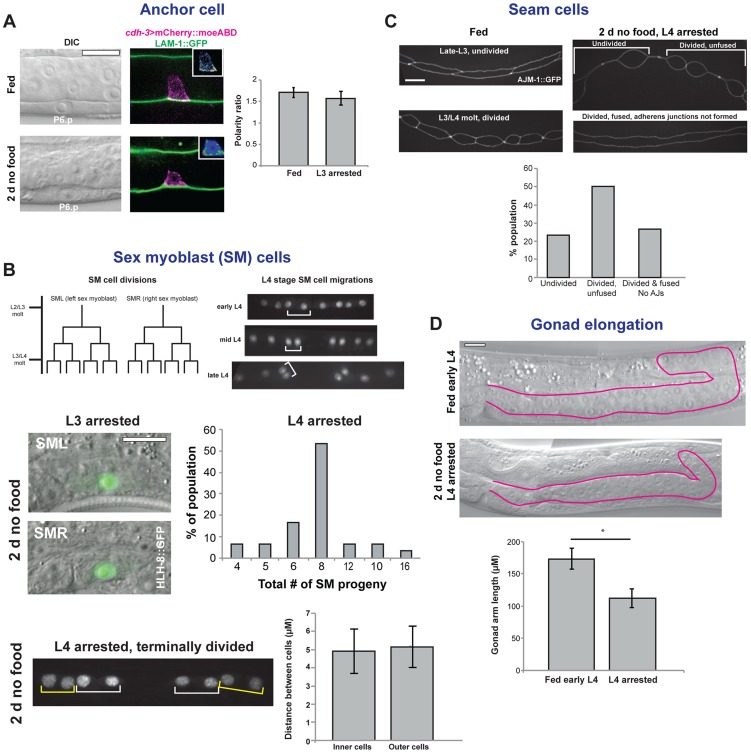
Arrest of tissues in the L3 and L4 stages. (A) Anchor cell (AC) arrest. Both fed and nutrient-deprived animals showed polarization of the AC-specific F-actin probe *cdh-3*>mCherry::moesinABD. Insets show heat maps of *cdh-3*>mCherry::moesinABD. Chart is quantification of polarity in fed and nutrient-deprived groups. Error bars ± S.E.M.; n = 16 per group. (B) Sex myoblast (SM) arrest. The SM cells divide three times between the mid L3 and early L4 stages, as depicted in the cell lineage diagram. Following cell divisions, the progeny cells (visualized with an HLH-8::GFP reporter gene) undergo short-range migrations during the L4 stage. In L3-arrested animals, no cell divisions occurred (n = 30). In animals removed from food in mid L3 that had arrested in early L4, two rounds of cell divisions typically occurred, although variability was present in the population. Graph shows percentage of the population with the indicated number of SM cell progeny (n = 30). When animals were grown to later times in L3 to allow completion of cell divisions, the short-range cell migrations that occur during the L4 stage were not observed. Chart compares distances between the nuclei of the two inner cells from each group of four (white brackets), which move closer together during the L4 stage, with the distance between the nuclei of the two outer cells (yellow brackets), which move further away from each other during L4. Error bars ± S.D.; n = 20 per group; *p* = .36 by two-tailed Student's *t*-test. (C) Seam cell arrest. Seam cells, which are separated by adherens junctions, divide during the molt, followed by fusion of the anterior daughter cell and re-formation of adherens junctions early in the larval stage. When animals were removed from food in the L3 stage and examined after 2 d, seam cells showed a variable pattern of arrest. In the top image, the posterior seam cells have divided but the anterior cells have not. In the bottom image, seam cells have divided and anterior daughter cells have fused, but the adherens junctions that separate cells have not re-formed. Chart shows quantification of arrested state in the V1 seam cell 2 d after food removal (n = 30). Seam cells were visualized with an AJM-1::GFP reporter gene. (D) Gonad elongation arrest. One of two gonad arms, outlined in magenta, in a fed early L4 animal and in an animal removed from food in the mid L3 stage. n = 20 animals; error bars ± S.D.; *p<1×10^−10^ by two-tailed Student's *t*-test. Scale bars, 10 µm.

Our experiments with vulval formation and the molting cycle showed that checkpoints are present early in the L3 and L4 stages that limit continued development. We took advantage of the fact that several additional tissues undergo developmental processes in the L3 and L4 stages to determine if other tissues are also arrested at the checkpoints. We first examined the uterine AC, which becomes polarized early in the L3 stage, when F-actin and actin regulators localize to the basal, invasive cell membrane [49]. The AC breaches the basement membrane in the mid L3 stage, before fusing with the surrounding uterine cells in the mid L4 stage (Fig. 1A). Examination of a marker of AC polarization, the F-actin probe *cdh-3*>mCherry::moesinABD, showed that animals removed from food late in the L2 stage arrested early in the L3 stage with polarized ACs, but in no instance did invasion occur (n = 100; Fig. 8A). When removed from food in the mid L3 stage, AC invasion occurred in all animals, yet in no instance did the AC fuse with the surrounding uterine cells (n = 100; Fig. 2B). These results suggest that the developmental program of the AC, similar to that the vulval cells and molting cycle, arrests at the early L3 and early L4 checkpoints.

We next examined the two sex myoblast (SM) cells, which divide three times between the mid L3 and early L4 stages, followed by short-range migrations of terminally divided progeny cells in the L4 stage (Fig. 8B). When animals were removed from food late in the L2 stage, no SM cell divisions were observed after 48 h, indicative of arrest early in the L3 stage. When removed from food in the mid L3 stage, SM cell divisions initiated in all animals and typically divided twice, although in some instances fewer or more cell divisions were observed (Fig. 8B). Animals that were grown on food to late in the L3 stage and arrested in the L4 stage with completed cell divisions did not undergo short-range migrations (Fig. 8B), demonstrating that the L4 morphogenetic program did not advance past the early L4 checkpoint. Although cell divisions of the SM cells are not as tightly regulated as the vulval cells, these results suggest that development of the SM cells is also under the control of the L3 and L4 checkpoints.

We then examined the seam cells, stem cells that divide during the L1–L3 molts, generating an anterior daughter that fuses with the surrounding hypodermal syncytium and a posterior daughter that retains stem-like properties (Fig. 8C). Animals that were removed from food in the L3 stage showed variability in the timing of seam cell arrest. Some seam cells failed to divide; others divided but anterior daughters did not fuse; and in the most advanced animals, daughter cells fused but the adherens junctions that connect seam cells did not re-form (Fig. 8C). Thus, removal of animals from food in the L3 stage causes the arrest of a several aspects of the seam cell division program prior to reaching the L4 checkpoint.

We finally looked at elongation of the gonad, which occurs in a continuous manner from the L2 to L4 stages. When animals were removed from food in mid L3 to cause arrested in the early L4 stage, gonad arm elongation was 35% shorter than in a fed control population of early L4 animals (Fig. 8D), indicating that gonadal elongation arrested prior to the L4 checkpoint. Taken together, these results show that diverse cellular processes arrest following removal of food. Although some tissues had a variable pattern of arrest, in no instance did development continue past the early L3 and early L4 stages, demonstrating that the checkpoints limit tissue development in a systemic manner.

## Discussion

**Figure 9:**
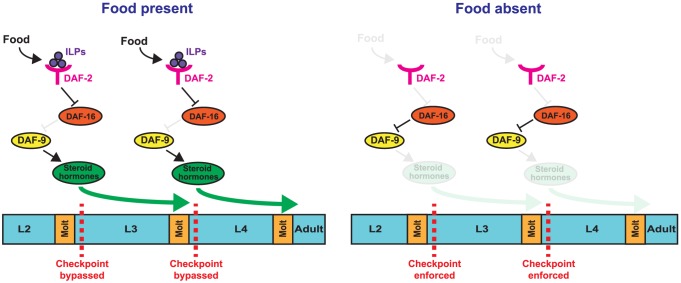
Model for nutritional regulation of L3 and L4 larval stage progressions. (Top) Feeding leads to the generation of insulin-like peptides (ILPs) that bind to the DAF-2 receptor, leading to the inhibition of DAF-16 activity. Inhibition of DAF-16 promotes expression of DAF-9 and the generation and release of steroid hormones, which allow animals to bypass the early L3 and early L4 checkpoints. (Bottom) In the absence of food, DAF-2 no longer inhibits DAF-16, which suppresses the expression of DAF-9 and the release of steroid hormones, causing animals to arrest at the checkpoints.

Organisms have the ability to sense their nutritional environment and alter growth and metabolism in response. When nutritional conditions are limiting during development, the effects on tissue maturation must be systemic in nature and temporally coordinated in order to maintain the capacity to form functional organs [1]. We examined the *C. elegans* response to nutrient deprivation during the L3 and L4 larval stages and have uncovered a means by which different tissues are able to arrest in a coordinated manner. We show that distinct checkpoints are present in the early part of the larval stages that regulate development throughout the organism and arrest a range of tissues and cellular processes. At the L3 and L4 checkpoints, nutritional conditions inform a systemic decision to either remain arrested or continue development. This decision is regulated by insulin-like and steroid hormone signaling pathways (see model, Fig. 9).

### Characterization of two new developmental checkpoints in the early L3 and early L4 larval stages

Previous work on L1 arrest, dauer, and adult reproductive diapause have shown that, in response to unfavorable nutritional conditions, cellular processes can arrest for extended durations and resume upon re-feeding [3], [7], [9], [10], [16]. The nature of the response to nutrient deprivation at other times in development had not been characterized. By focusing initially on the vulva, which has a stereotyped pattern of development during the L3 and L4 larval stages, we show here that checkpoints are present in the early part of the L3 and L4 stages that arrest tissues throughout the organism. The timing of arrest reflects a specific point in the larval stage after molting and prior to initiation of the subsequent molting cycle. A connection between nutrition and the molting cycle has been described in other ecdysozoans [50], [51]. In insects, for instance, molting to a new larval instar occurs only after a sufficient duration of feeding and attainment of a critical weight [50]. It has been speculated that similar nutritional factors impinge on the endocrine signals that trigger the onset of the molting cycle in *C. elegans* [38]. Our results provide support for this model of nutritional control of molting cycle commitment.

The response to nutrient deprivation in the L3 and L4 stages is systemic in nature and causes the arrest of multiple tissues and cellular processes. Although all tissues arrested prior to or at the checkpoints, vulval formation and the molting cycle were unique in arresting within very narrow developmental windows and in a uniform manner throughout a population. These tissues may have been under selection to arrest in such a precise way. A properly formed vulva is necessary for mating and egg-laying, and a robust developmental program with minimal variation is important for reproductive fitness [52]. For the molting cycle, the inability to shed cuticle surrounding the head during ecdysis causes rapid lethality, as observed in *daf-9*–overexpressing animals, making it incumbent to execute the molt. A previous study showed that the buccal cavity, which comprises the anterior-most portion of the pharynx and constrains the amount of food consumed with each pumping event, grows only during molts and not between them, which is thought to increase the amount of food that can be consumed during the larval stage [53]. Certain tissues therefore have distinct patterns of growth that are unique to their functions in development and reproduction.

### The insulin-like and DAF-9 signaling pathways regulate arrest following nutrient deprivation

We show that the insulin-like signaling pathway regulates that connection between nutritional conditions and progression past the L3 and L4 checkpoints. In wild type animals, feeding of 30–60 minutes is required after molting to attain a sufficient threshold for bypassing the larval stage checkpoints. Perturbations of key genes in the insulin-like signaling pathway alter the duration of feeding required to bypass arrest. Reduction in the function of *daf-2*, the insulin/IGF receptor, increases the amount of feeding, such that animals pause at the L3 and L4 checkpoints and have delayed development through the L3 and L4 stages. Loss of function of *daf-16*, a FOXO transcription factor that is a key target of the insulin-like signaling pathway [54], decreases the amount of feeding required to bypass the checkpoints.

The bypass of arrest caused by loss of *daf-16* is partially suppressed by reduced expression of *daf-9*, a cytochrome P450 ortholog required for the production of certain *C. elegans* steroid hormones [18], [55]. This result suggests that DAF-16 regulates arrest in the L3 and L4 stages at least in part through inhibition of steroid hormone signaling. DAF-16 has been shown to inhibit *daf-9* expression during cholesterol starvation, an unfavorable growth environment that causes larval arrest [32], [56]. It is possible that late larval stage arrest caused by nutrient deprivation also involves DAF-16 inhibition of *daf-9* expression. A key site of action for DAF-16 in regulating L3 and L4 arrest is the hypodermis, where *daf-9* is also expressed during larval development [47], further suggesting that DAF-16 regulates *daf-9* expression. Collectively, these findings support a model in which DAF-16 inhibits *daf-9* expression, and possibly other genes involved in steroid hormone production, to limit progression through the L3 and L4 stages (Fig. 9).

The level of DAF-9 protein is a key determinant of L3 and L4 stage progressions in the absence of food, which is demonstrated by the striking ability of overexpressed DAF-9 to promote continued development through one or two larval stages. The hormonal signaling pathway that functions downstream of DAF-9 in the L3 and L4 stages appears to be different from the pathway that regulates dauer formation. During the dauer decision, the DAF-9 biosynthetic pathway produces dafachronic acids (DAs), which bind to the nuclear hormone receptor DAF-12 to promote bypass of dauer [18]. Our experiments with a *daf-12* null mutant failed to show a similar role for DAF-12 in the L3 and L4 stages. Consistent with these results, we have also found that supplementation of M9 buffer with DAs does not promote continued development past the checkpoints after food removal (Schindler & Sherwood, unpublished observations), further implicating a mode of hormone signaling during the L3 and L4 larval stages that is distinct from that during dauer formation.

### A developmental decision regulates progression through the L3 and L4 larval stages

A key implication from these results is that wild type *C. elegans* arrest development in the L3 and L4 stages despite possessing a sufficient amount of nutrients to continue further development. This is demonstrated by the ability of animals lacking *daf-16* or overexpressing *daf-9* to bypass one or even two arrest points and progress through the larval stages in the absence of food. Developmental arrest in wild type animals therefore reflects a decision to halt larval stage progressions rather than a lack of available resources to sustain further development. Continued progression in the absence of food appears to have deleterious consequences, as exemplified by the death and molting defects observed in *daf-9*–overexpressing animals. Limiting progression through the larval stages when nutritional conditions are poor may allow resources to be conserved for survival and tissue homeostasis during prolonged periods of starvation.

This scenario of sensing the environment and arresting development in response to unfavorable conditions also occurs during the *C. elegans* dauer decision [3]. Both non-dauer and dauer arrest are regulated by insulin-like and DAF-9 signaling pathways, and studies comparing gene expression in dauer and starved animals have revealed overlap between the two types of arrest [57], [58]. From an evolutionary perspective, it is intriguing to speculate that dauer formation, a nematode-specific developmental diapause, evolved from pathways of starvation-induced arrest that are conserved among metazoans.

### A mode of saltational growth regulates *C. elegans* development

Two types of growth have been described for ecdysozoans: continuous, with growth occurring throughout the course of development; and saltational, with growth occurring only at distinct times [53]. In organisms with rigid exoskeletons, growth occurs only at molts, an example of saltational growth. *C. elegans*, with flexible exoskeletons, grow in a continuous manner through the larval stages [53], [59]. By manipulating the nutritional environment, we show that *C. elegans* growth has an additional saltational aspect to it, with distinct checkpoints present in the early part of the larval stage. At each checkpoint the nutritional environment informs a systemic decision to either proceed through the larval stage or to remain arrested (Fig. 9). Two key pathways that regulate this developmental decision—insulin-like and steroid hormone signaling—are present throughout metazoans [60], [61], suggesting that the mode of growth control described in this work could be conserved. A greater understanding of the mechanisms of growth control could provide insight into aging and metabolic diseases, which are linked to the dysregulation of developmental pathways important for growth [62], [63]. Our work in *C. elegans* demonstrates a type of saltational growth from checkpoint to checkpoint that may similarly regulate development and physiology in other species.

## Materials and Methods

### General methods and strains

Nematodes were reared at 20°C on NGM plates seeded with OP50 *E. coli* using standard procedures. In the text and figures we refer to linked DNA sequences that code for a single fusion protein using a (::) annotation. For designating linkage to a promoter we use a (>) symbol. The wild type strain N2 and the following mutant strains and transgenes used were: *dhIs64*[*daf-9::GFP*], *qyEx262*[*unc-119>GFP::daf-16*], *qyEx263*[*daf-16>GFP::daf-16*], *qyEx264*[*myo-2>GFP::daf-16*], *qyEx266*[*GFP::daf-16*], *qyEx267*[*ges-1>GFP::daf-16*], *qyEx268*[*unc-115>GFP::daf-16*], *qyEx292*[*col-12>GFP::daf-16*], *kbIs7*[*nhx-2>rde-1*], *kzIs9*[*lin-26>rde-1*], *kzIs20*[*hlh-1>rde-1*]; LG I: *daf-16(mu86)*, *ayIs4*[*egl-17*>GFP], *syIs78*[*ajm-1::GFP*]; LGII: *qyIs17*[*zmp-1>mcherry*]; LG III: *daf-2(e1370)*, *unc-119(ed4)*; *glp-1(e2144); zhIs4*[*lip-1>NLS-GFP*]; LG IV: *mgIs49*[*mlt-10>GFP-PEST*], *ayIs7*[*hlh-8::GFP*], *qyIs10*[*lam-1::GFP*]; LG V: *rde-1*(*ne219*), *qyIs50*[*cdh-3>mCherry::moesinABD*]; LG X: *hbl-1(ve18)*, *qyIs7*[*lam-1::GFP*]; *daf-12(rh61rh411)*.

### Image acquisition, processing and data analysis

Images were acquired using either a Zeiss AxioImager A1 microscope with a 10×, 20×, or 100× plan-apochromat objective and a Zeiss AxioCam MR charge-coupled device camera, controlled by Zeiss Axiovision software (Zeiss Microimaging, Inc., Thornwood, NJ), or with a Yokogawa spinning disk confocal microscope mounted on a Zeiss AxioImager A1 microscope using iVision software (Biovision Technologies, Exton, PA). Images were processed in ImageJ (NIH Image) and Photoshop CS6 (Adobe Systems Inc., San Jose, CA). Z-stack projections were generated using IMARIS 6.0 (Bitplane, Inc., Saint Paul, MN).

Quantification of fluorescence intensity was performed on images acquired at identical exposure settings using ImageJ. For quantifying GFP::DAF-16 in the hypodermis, the fluorescence intensity in four nuclei (excluding nucleoli) were averaged per animal. All measurement of nuclear GFP::DAF-16 were taken within 5 min of removal from food to minimize relocalization of DAF-16 into the nucleus. For quantifying DAF-9::GFP, a contiguous area of the hypodermal syncytium that excluded nuclei was measured in a region below the pharynx.

### Food removal assays

Populations containing gravid adults were hypochlorite treated to release embryos, which hatched in M9 buffer and arrested in L1. The duration of L1 arrest did not exceed 20 h. Populations of L1-arrested animals were plated onto NGM plates seeded with OP50 *E. coli* that covered at least half the plate to minimize the duration of wandering away from food. Maximum population density was 2500 animals/60 mm dish. Animals were reared at 20°C unless indicated otherwise. For removal from food late in the L2 stage, populations were monitored starting at 22 h post-plating. The assessment of developmental age was made by observation of the gonad (which grows through the L2 stage) and the molt (which covers the mouth during the time of molting, see Fig. 1A). Unless a specific duration of growth is indicated, animals were removed from food when the oldest members of the population were molting, and the remaining members were in the late L2 stage, based on gonad size. Populations that contained greater than 5% L3 animals were not used. N2 and *daf-12(rh61rh411)* populations typically developed in a synchronized manner; *hbl-1(ve18)*, *daf-16(mu86)*, and *daf-9::GFP*, populations grew more variably and had a wider spread of developmental ages at the time of food removal. To remove food, 1 ml M9 was added to each plate and gently rocked to dislodge worms with minimal removal of *E. coli*. Animals were transferred to low-retention Eppendorf tubes and centrifuged for 1 min at 500× g, a speed at which *C. elegans* sank to the bottom but *E. coli* remained largely in suspension. Liquid was aspirated, and an additional 1 ml M9 added for 6 total washes. Tests of supernatants found that bacteria were removed by the third wash, based on the inability of the supernatant to form colonies on LB plates. Animals were placed in M9 buffer at 0.5–1 animal/µl in 25-ml glass conical tubes and rotated in a roller drum (New Brunswick Scientific, Enfield, CT) at ambient temperature (22°C). For visualization of ecdysis, animals were removed from food late in the L2 stage, anesthetized in levamisole, mounted on agar pads with sealed cover slips, and maintained for 24 h in a humidified chamber.

### Scoring developmental stages

Developmental stages from L3 to young adult were assessed using the progression of the vulva (see Fig. 1A) and the molt. The two processes occurred synchronously in both fed and nutrient-deprived animals. Statistical significance of differences in arrest response was determined by two-tailed Fisher's exact test. For tissue-specific *daf-16* rescue experiments, percentages of L3- and L4-arrested animals were determined for each promoter-driven *GFP::daf-16* strains and compared to the promoterless *GFP::daf-16* strain (*qyEx266*). For tissue-specific *daf-16* dsRNA feeding, percentages of L3- and L4-arrested animals were compared between animals fed either *daf-16* dsRNA or vector control. A similar comparison was made in *daf-16(mu86)* animals fed either *daf-9* dsRNA or vector control. All assays were repeated in triplicate with n≥50 animals per assay.

### Survival and recovery assays

Populations of wild type animals were removed from food in either late in the L2 or in the middle of the L3 stage and starved in M9 buffer at an approximate population density of 1 animal/µl. Every 2 d, an aliquot of media containing at least 50 animals was removed using Rainex-coated tips to prevent adherence to the plastic, and plated onto NGM plates. After liquid was absorbed into the plate, animals were determined to be alive if they moved or dead if they did not move upon tail poke. Dead animals had a characteristic rod-like appearance. The median survival was determined as the first day at which 50% of the population was dead, for n = 3 trials.

To test recovery from nutrient deprivation, early L3 arrested animals were plated onto NGM+OP50 after 8 d in the absence of food. After 72 h at 20°C, the population was scored for fertile and nonfertile adults.

### Determination of feeding requirements to bypass arrest

Synchronous populations were grown to the L2/L3 or L3/L4 molts. Animals in ecdysis were isolated by the appearance of detached cuticle separated from the body, which was most apparent in the head and tail regions. Individual animals were transferred onto NGM+OP50 plates for 30′–90′ further feeding or placed directly in M9 buffer. Animals were maintained in the absence of food for 24 h and the developmental stage assessed.

### Generation of *GFP::daf-16* transgenic strains

Transgenic strains expressing promoter-driven *daf-16* cDNA fused at the N-terminus with GFP were generated by injection of the following plasmids: pNL205 (promoterless), pNL206 (*unc-119* promoter), pNL209 (*daf-16* promoter), pNL212 (*myo-3* promoter), pNL213 (*ges-1* promoter), pNL216 (*unc-115* promoter), and pAS10 (*col-12* promoter). With the exception of pAS10, plasmids were gifts of the Kenyon lab and are described elsewhere [42]. pAS10 was generated by PCR amplification of 1.1 kb of *col-12* promoter 5′ to the start site, which was cloned into the SnaBI restriction sites in pNL205. *GFP::daf-16* plasmids were injected at 100 ng/µl into *daf-16(mu86); unc-119(ed4)* adults with 50 ng/µl *unc-119*(+) plasmid. Animals carrying extrachromosomal arrays were isolated by rescue of the *unc-119* locomotion defect and the expression of GFP::DAF-16 validated. Although *unc-115* has been reported to express in both neurons and hypodermis [64], expression was only detected in neurons. With the exception of *qyEx266* (expressing pNL205), which did not possess a gene promoter for GFP::DAF-16, and *qyEx292* (expressing pAS10), which sometimes had undetectable or minimal GFP expression in the absence of food, animals carrying the array were identified in nutrient removal assays by GFP expression. *qyEx266* and *qyEx292* animals were plated on NGM plates lacking food, and those that moved freely (indicating presence of the *unc-119* rescue array) were selected for analysis.

### Tissue-specific RNAi

The generation and validation of strains sensitive to RNAi in the hypodermis (NR222, *rde-1(ne219)*; *lin-26>rde-1*); muscle (NR350, *rde-1(ne219)*; *hlh-1>rde-1*); and intestine (VP303, *rde-1(ne219)*; *nhx-2>rde-1*) are described elsewhere [30], [65]. Strains were fed either *daf-16* or L4440 (vector control) dsRNA starting from L1 arrest, grown to late in the L2 stage, and removed from food. After 2 d in the absence of food, the developmental stage was scored.

### Measurements of AC polarity, SM cell distance, and gonad elongation

To assess AC polarization, the average fluorescence intensity was determined from three-pixel-wide linescans drawn along either the basal or apicolateral membranes of Z-stack projections. To determine the movement of SM cell progeny, the distance between the nuclei of the two inner cells from among the four cells on each lateral half were measured. A similar measurement was made to determine the distance between the two outer nuclei. Gonad length was measured from the vulva to the distal end. All measurements were made using ImageJ software.

## Supporting Information

Figure S1 Raw data and replicate assays of wild type time course experiments. The graphs in Figs. 1B, 2A, and 2C are reproduced to show the percentages of animals and sample sizes at each time point. Percentages are rounded to the nearest whole number and may not equal 100. To the right are results of three replicate assays, with measurements at 24, 48, and 96 h. The percentage of animals at each developmental stage is averaged across the three experiments. Error bars+S.D.
(PDF)

Figure S2 Nutrition-induced L3 arrest is different from dauer arrest. L3-arrested animals are similar in length to animals in dauer, but do not undergo radial constriction (body narrowing). Inset shows hypodermis, which forms alae (indicated with arrows) in dauers but not in L3-arrested animals. Scale bar, 100 µM; inset scale bar, 10 µM.
(PDF)

Figure S3 Arrest in L4 occurs at a precise time in vulval development. Cell-cell fusions occur between homotypic cells following terminal vulval cell divisions. Shown on left is a lateral schematic of the vulva after terminal cell divisions. This view was rotated 90°C to a distal-proximal view (from tail to midbody), and one-half of the vulva is shown. Schematics show that the vulA cells on each lateral half of the vulval midline fuse at approximately 35 h post-hatch, followed 2 h later by vulC cell fusion [33]. Animals removed from food after 28 h (mid L3 stage) undergo vulA fusions but not vulC fusions (detectable by the presence of a membrane boundary between cells, yellow arrowhead). When animals were removed from food after 32 h (L3/L4 molt), 97% of the population arrested at the same stage of cell-cell fusions, although VPCs migrated further toward the midline (n = 30 per assay). Membranes are demarcated by the apical junction reporter gene AJM-1::GFP. Scale bars, 3 µM.
(PDF)

Figure S4 Feeding is required after molting to bypass the L3 and L4 checkpoints. A schematic of the *C. elegans* larval stage is depicted on the left. Ecdysis, the shedding of cuticle, occurs after the period of lethargus and precedes foraging. Animals were removed from food at ecdysis or at 30 min intervals thereafter, and assayed for developmental stage after 24 h. Results show that approximately 30–60 min feeding is required after molting to bypass developmental checkpoints in the L3 and L4 stages.
(PDF)

Figure S5 Raw data and replicate assays of *daf-16(mu86)* and DAF-9::GFP time course experiments. The graphs in Figs. 5A, 5B, and 7B are reproduced to show the percentages of animals and sample sizes at each time point. Percentages are rounded to the nearest whole number and may not equal 100. To the right are results of three replicate assays, with measurements at 24, 48, and 96 h. The percentage of animals at each developmental stage is averaged across the three experiments. Error bars+S.D.
(PDF)

Figure S6 Expression patterns of GFP::DAF-16 transgenic strains. Promoterless GFP::DAF-16 has no detectable expression (signal is due to autofluorescence). *daf-16*>GFP::DAF-16 had prominent expression in neurons (N), hypodermis (Hyp), body wall muscle (BWM), and intestine (Int). Tissue-specific strains expressed in the predicted tissues: *unc-119* and *unc-115* promoter constructs in the neurons, *myo-3* in the body wall muscle, *ges-1* in the intestine, and *col-12* in the hypodermis. All bottom images taken at 500 ms exposure. Scale bar top images, 100 µM; bottom images, 10 µM.
(PDF)

Figure S7 Expression of *col-12*>GFP::DAF-16 declines following removal from food compared to *daf-16*>GFP::DAF-16. Fluorescence intensity measurements were taken of hypodermal nuclei in feeding L3 stage animals and 1–3 d after removal from food. Error bars ± S.E.M.; n = 25 animals for each measurement.
(PDF)
