# Supplementary figures and images for: Identification of Late Larval Stage Developmental Checkpoints in Caenorhabditis elegans Regulated by Insulin/IGF and Steroid Hormone Signaling Pathways
Source: PLoS Genet. 2014 Jun 19;10(6):e1004426. doi: 10.1371/journal.pgen.1004426 (PMC4063711; doi:10.1371/journal.pgen.1004426)

Figure S1

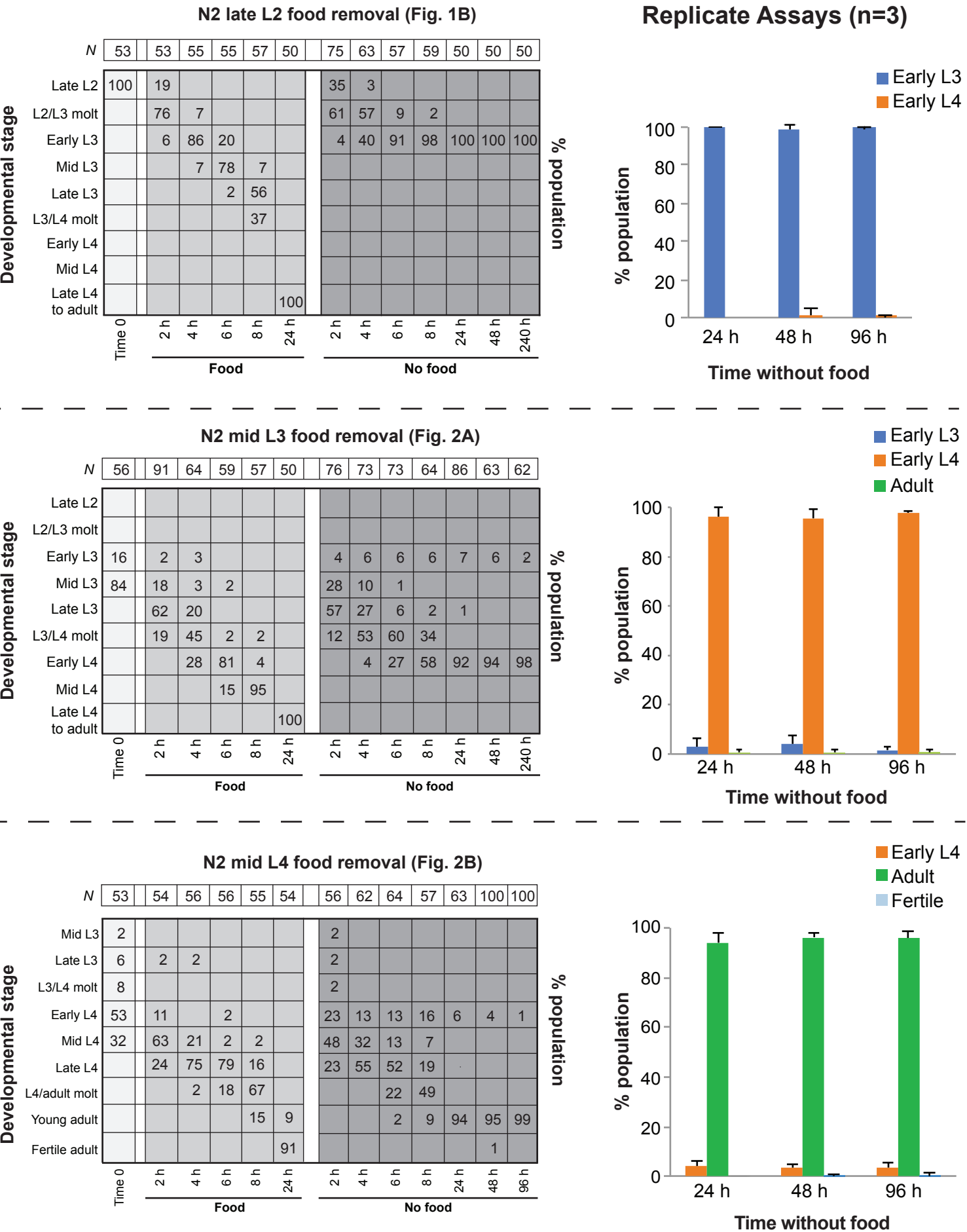

Supplement: Figure S1 — Raw data and replicate assays of wild type time course experiments. The graphs in Figs. 1B, 2A, and 2C are reproduced to show the percentages of animals and sample sizes at each time point. Percentages are rounded to the nearest whole number and may not equal 100. To the right are results of three replicate assays, with measurements at 24, 48, and 96 h. The percentage of animals at each developmental stage is averaged across the three experiments. Error bars+S.D. (PDF) [file pgen.1004426.s001.pdf]

Figure S2

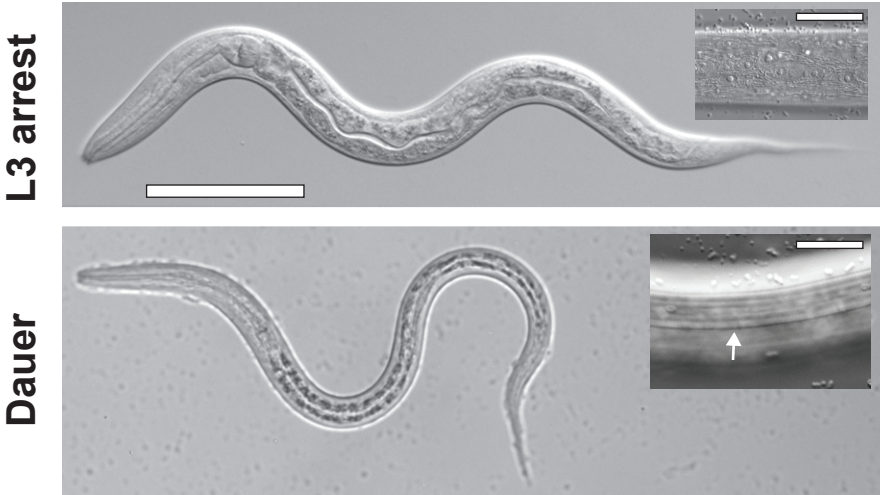

Supplement: Figure S2 — Nutrition-induced L3 arrest is different from dauer arrest. L3-arrested animals are similar in length to animals in dauer, but do not undergo radial constriction (body narrowing). Inset shows hypodermis, which forms alae (indicated with arrows) in dauers but not in L3-arrested animals. Scale bar, 100 µM; inset scale bar, 10 µM. (PDF) [file pgen.1004426.s002.pdf]

**Figure S3**

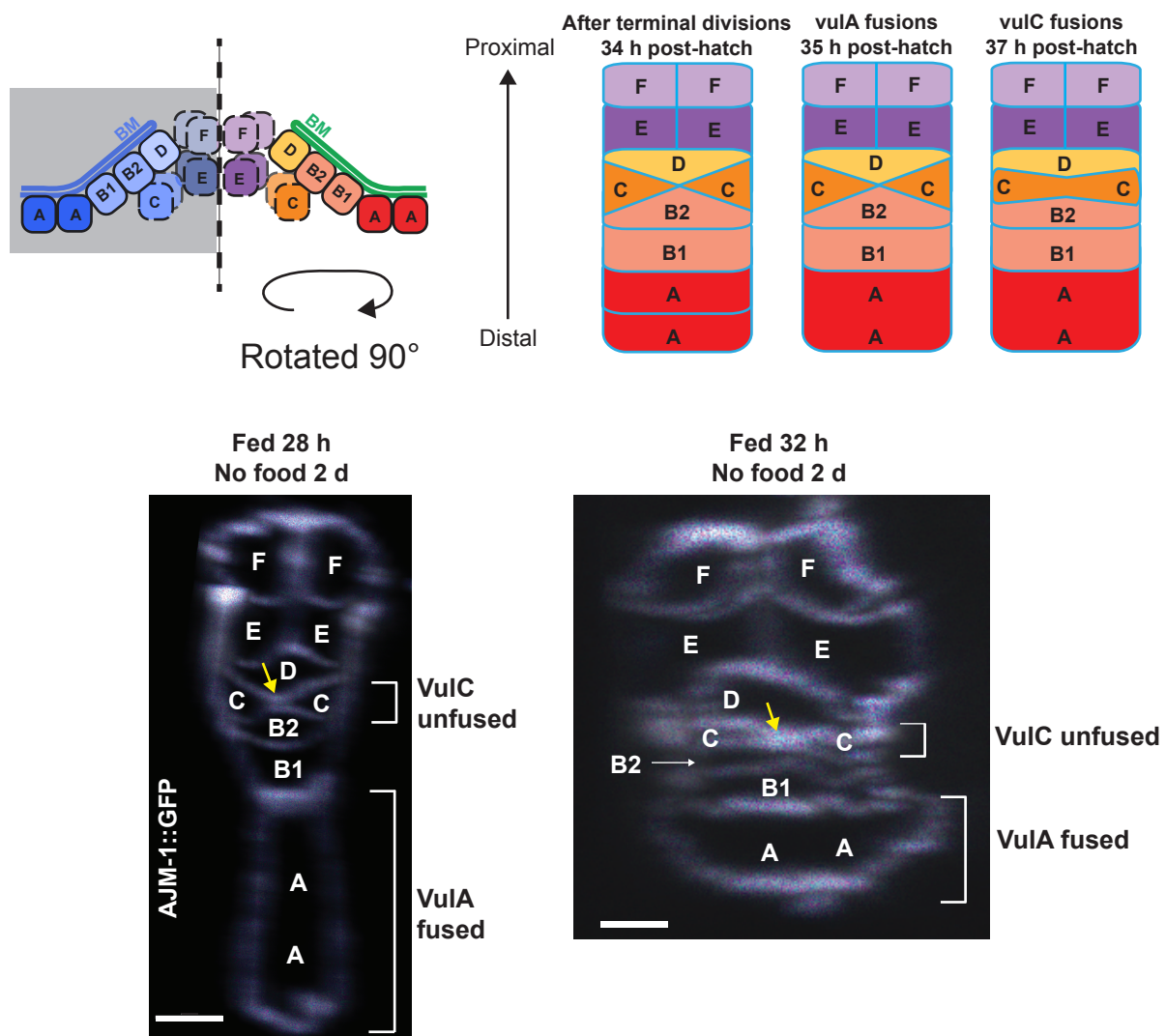

Supplement: Figure S3 — Arrest in L4 occurs at a precise time in vulval development. Cell-cell fusions occur between homotypic cells following terminal vulval cell divisions. Shown on left is a lateral schematic of the vulva after terminal cell divisions. This view was rotated 90°C to a distal-proximal view (from tail to midbody), and one-half of the vulva is shown. Schematics show that the vulA cells on each lateral half of the vulval midline fuse at approximately 35 h post-hatch, followed 2 h later by vulC cell fusion [33]. Animals removed from food after 28 h (mid L3 stage) undergo vulA fusions but not vulC fusions (detectable by the presence of a membrane boundary between cells, yellow arrowhead). When animals were removed from food after 32 h (L3/L4 molt), 97% of the population arrested at the same stage of cell-cell fusions, although VPCs migrated further toward the midline (n = 30 per assay). Membranes are demarcated by the apical junction reporter gene AJM-1::GFP. Scale bars, 3 µM. (PDF) [file pgen.1004426.s003.pdf]

Figure S4

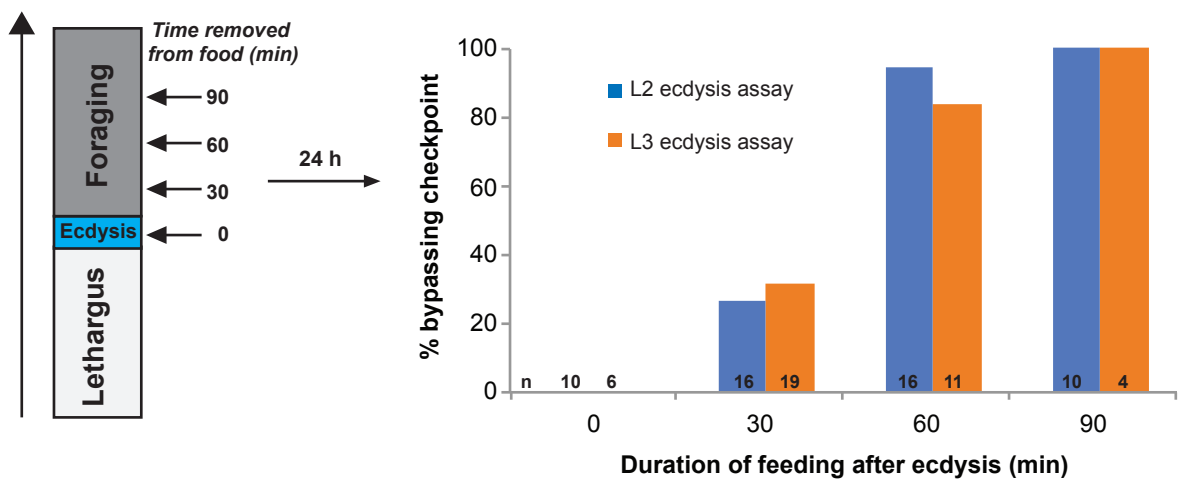

Supplement: Figure S4 — Feeding is required after molting to bypass the L3 and L4 checkpoints. A schematic of the C. elegans larval stage is depicted on the left. Ecdysis, the shedding of cuticle, occurs after the period of lethargus and precedes foraging. Animals were removed from food at ecdysis or at 30 min intervals thereafter, and assayed for developmental stage after 24 h. Results show that approximately 30–60 min feeding is required after molting to bypass developmental checkpoints in the L3 and L4 stages. (PDF) [file pgen.1004426.s004.pdf]

Figure S5

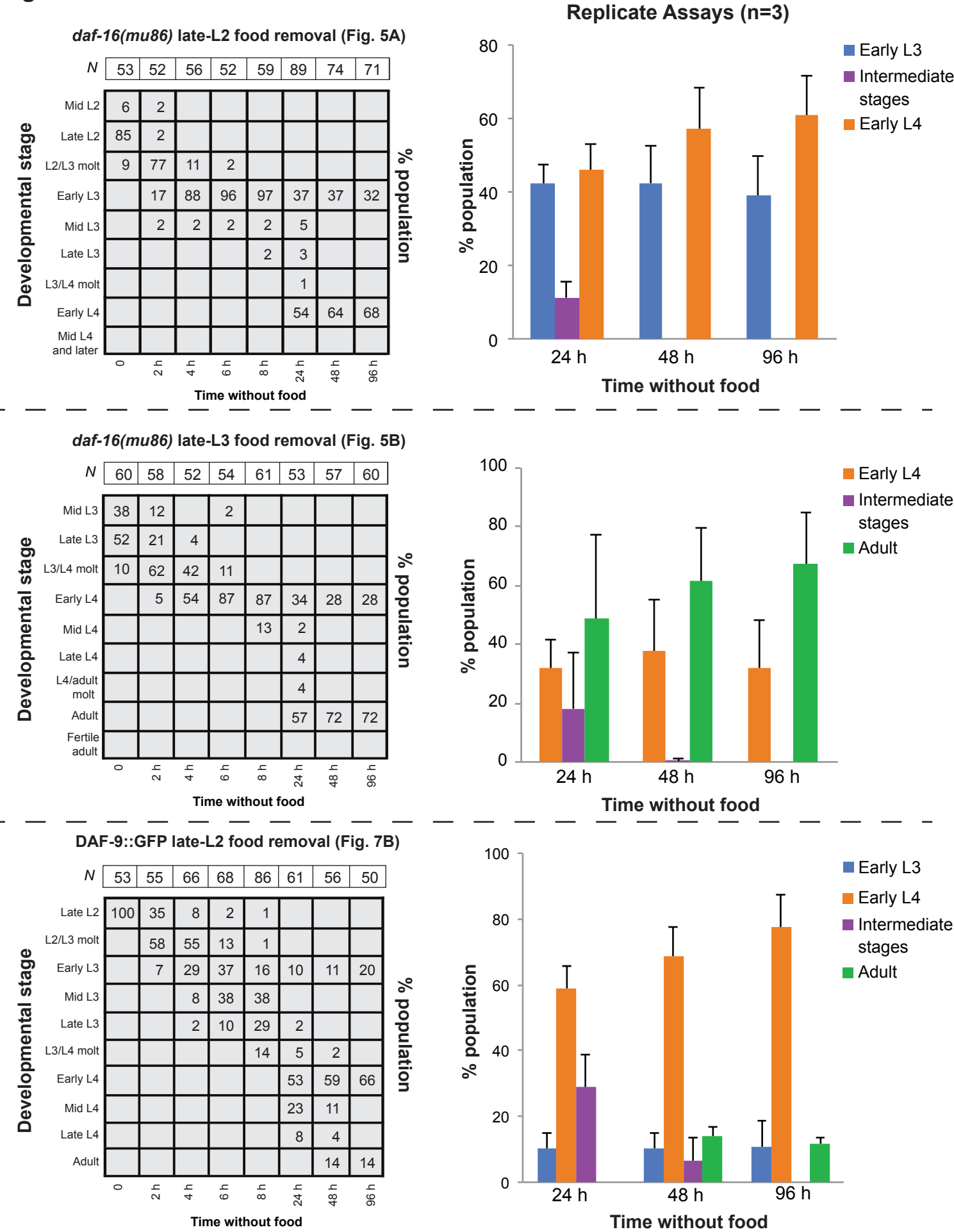

Supplement: Figure S5 — Raw data and replicate assays of daf-16(mu86) and DAF-9::GFP time course experiments. The graphs in Figs. 5A, 5B, and 7B are reproduced to show the percentages of animals and sample sizes at each time point. Percentages are rounded to the nearest whole number and may not equal 100. To the right are results of three replicate assays, with measurements at 24, 48, and 96 h. The percentage of animals at each developmental stage is averaged across the three experiments. Error bars+S.D. (PDF) [file pgen.1004426.s005.pdf]

Figure S6

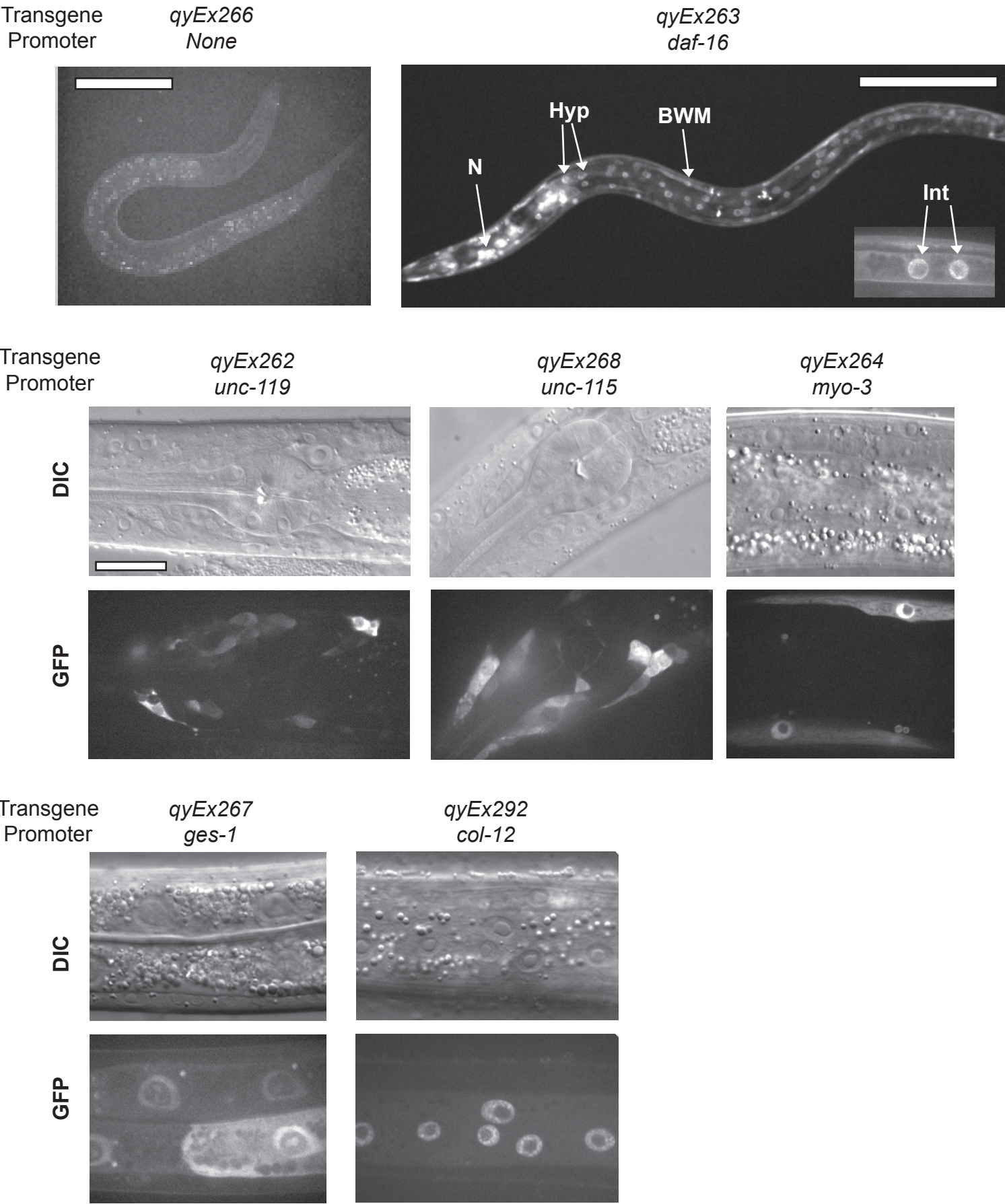

Supplement: Figure S6 — Expression patterns of GFP::DAF-16 transgenic strains. Promoterless GFP::DAF-16 has no detectable expression (signal is due to autofluorescence). daf-16>GFP::DAF-16 had prominent expression in neurons (N), hypodermis (Hyp), body wall muscle (BWM), and intestine (Int). Tissue-specific strains expressed in the predicted tissues: unc-119 and unc-115 promoter constructs in the neurons, myo-3 in the body wall muscle, ges-1 in the intestine, and col-12 in the hypodermis. All bottom images taken at 500 ms exposure. Scale bar top images, 100 µM; bottom images, 10 µM. (PDF) [file pgen.1004426.s006.pdf]

Figure S7

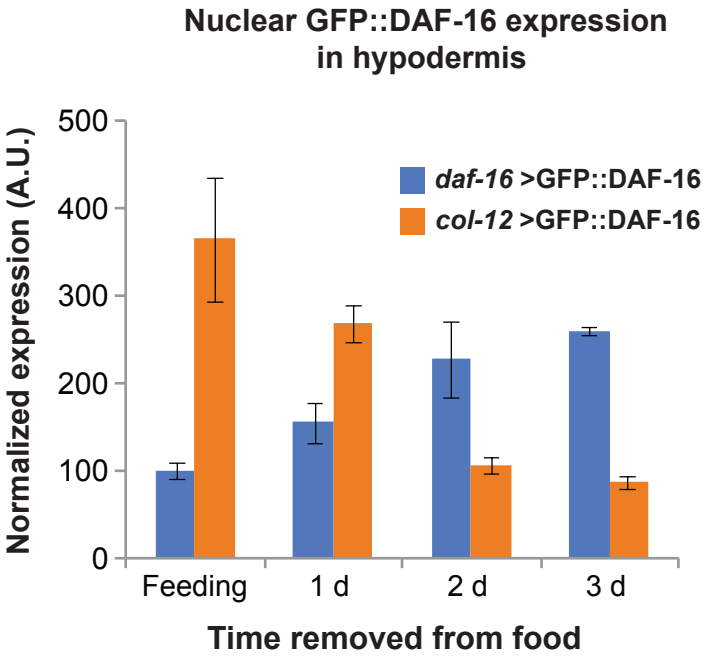

Supplement: Figure S7 — Expression of col-12>GFP::DAF-16 declines following removal from food compared to daf-16>GFP::DAF-16. Fluorescence intensity measurements were taken of hypodermal nuclei in feeding L3 stage animals and 1–3 d after removal from food. Error bars ± S.E.M.; n = 25 animals for each measurement. (PDF) [file pgen.1004426.s007.pdf]
